# Causal associations of Sjögren’s syndrome with cancers: a two-sample Mendelian randomization study

**DOI:** 10.1186/s13075-023-03157-w

**Published:** 2023-09-15

**Authors:** Yiwei Jia, Peizhuo Yao, Jia Li, Xinyu Wei, Xuanyu Liu, Huizi Wu, Weiwei Wang, Cong Feng, Chaofan Li, Yu Zhang, Yifan Cai, Shuqun Zhang, Xingcong Ma

**Affiliations:** Department of Oncology, the Second Affiliated Hospital of Xi’an Jiaotong University, Xi’an, Shaanxi 710004 People’s Republic of China

**Keywords:** Sjögren’s syndrome, Cancer risks, Mendelian randomization, Causality

## Abstract

**Background:**

Several observational studies have explored the associations between Sjögren’s syndrome (SS) and certain cancers. Nevertheless, the causal relationships remain unclear. Mendelian randomization (MR) method was used to investigate the causality between SS and different types of cancers.

**Methods:**

We conducted the two-sample Mendelian randomization with the public genome-wide association studies (GWASs) summary statistics in European population to evaluate the causality between SS and nine types of cancers. The sample size varies from 1080 to 372,373. The inverse variance weighted (IVW) method was used to estimate the causal effects. A Bonferroni-corrected threshold of *P* < 0.0031 was considered significant, and *P* value between 0.0031 and 0.05 was considered to be suggestive of an association. Sensitivity analysis was performed to validate the causality. Moreover, additional analysis was used to assess the associations between SS and well-accepted risk factors of cancers.

**Results:**

After correcting the heterogeneity and horizontal pleiotropy, the results indicated that patients with SS were significantly associated with an increased risk of lymphomas (odds ratio [OR] = 1.0010, 95% confidence interval [CI]: 1.0005–1.0015, *P* = 0.0002) and reduced risks of prostate cancer (OR = 0.9972, 95% CI: 0.9960–0.9985, *P* = 2.45 × 10^−5^) and endometrial cancer (OR = 0.9414, 95% CI: 0.9158–0.9676, *P* = 1.65 × 10^−5^). Suggestive associations were found in liver and bile duct cancer (OR = 0.9999, 95% CI: 0.9997–1.0000, *P* = 0.0291) and cancer of urinary tract (OR = 0.9996, 95% CI: 0.9992–1.0000, *P* = 0.0281). No causal effect of SS on other cancer types was detected. Additional MR analysis indicated that causal effects between SS and cancers were not mediated by the well-accepted risk factors of cancers. No evidence of the causal relationship was observed for cancers on SS.

**Conclusions:**

SS had significant causal relationships with lymphomas, prostate cancer, and endometrial cancer, and suggestive evidence of association was found in liver and bile duct cancer and cancer of urinary tract, indicating that SS may play a vital role in the incidence of these malignancies.

**Supplementary Information:**

The online version contains supplementary material available at 10.1186/s13075-023-03157-w.

## Introduction

Sjögren’s syndrome (SS) is a systemic chronic autoimmune disorder characterized by lymphocytic infiltration of the exocrine glands including salivary and lacrimal glands, which lead to significant loss of the secretory function [1]. SS is among the most common autoimmune diseases, along with systemic lupus erythematosus and progressive systemic sclerosis. Its overall prevalence varies from 0.1 to 4.8% [2]. It is more prevalent in women than in men (average female to male ratio 9:1), and the susceptible population are young women aged 20 s to 30 s and women after the menopause in the mid-50 s. SS can impact multiple organ systems including respiratory system, digestive system, urinary system, circulatory system, and nervous system which lead to different symptoms such as interstitial pneumonitis, tracheobronchial sicca, autoimmune hepatitis, primary biliary cholangitis, interstitial nephritis, pericarditis, pulmonary hypertension, and sensory neuropathies [1]. Clinically, accurate diagnosis and individual treatment for SS are often challenging due to the complexity of etiology and the diversity of manifestation among SS patients. The pathogenesis of SS can be multifactorial. Individuals with genetic predisposition are thought to develop SS through the effect of certain environmental factors; however, the underlying causes and detailed mechanisms remain unclear [3].

Epidemically, patients with Sjögren’s syndrome history seemed to be under higher risks for developing certain cancers. Several studies have investigated the potential connection between SS and different cancers, among which the association between SS and lymphomas was mostly studied. The results indicated that patients with SS tend to have a significantly higher lymphoma risk than healthy individuals [4, 5]. However, the associations between SS and other types of cancers were controversial. A prospective cohort study from Spain showed that SS is associated with the development of thyroid, oral cavity, and gastric cancer [6]. In a retrospective cohort study from Korea, in addition to non-Hodgkin lymphoma, SS patients also have an increased risk of solid cancers including oropharynx, thyroid, and lung cancers [7]. Another retrospective cohort study from England illustrated that patients with SS were not at higher risk for other cancers, such as lung cancer, breast cancer, ovarian cancer, pancreatic cancer, and melanoma, except for lymphoma [8]. Goulabchand et al. [9] found that SS patients were less likely to develop breast cancer and more likely to develop thyroid cancer observed in clinical cohort in France. Brom et al. [10] showed that compared to overall population, women in Argentina with SS were more likely to develop multiple myeloma, breast cancer, and tongue cancer.

The pathophysiological mechanisms about how SS may lead to certain malignancy, that is, whether there are causal effects of SS on cancers remains to be elucidated. Current researches suggests that shared genetic and environmental risk factors or process may contribute to oncogenesis among SS patients. The correlation between DNA methylation and disease progression in SS patients has been studied a lot. Major methylation alterations presenting in B cells and the genetic at-risk loci in SS patients were identified, and the methylation status of B cells was proved to be strongly correlated with disease progression [11]. Imgenberg-Kreuz et al. [12] found prominent hypomethylation of interferon (IFN) regulated genes in whole blood and CD19^+^ B cells, which resulted in increased expression of these genes. DNA methylation abnormalities are known to cause chromosomal instability, the activation of various oncogenes, or transcriptional inactivation of tumor suppressor genes, thus leading to oncogenesis [13, 14]. Wang et al. [15] identified a small cluster of differently expressed miRNA in SS including miR-146a-5p and miR-30b-5p. miR-146a is significantly upregulated in SS patients and is involved in the upregulation of phagocytic activity and in the reduction of inflammatory cytokine production [16]. miR-146a has been considered as either an oncogene or tumor suppressor gene dependent on the type of cancers [17–19]. miR-30b is inversely correlated with the expression of B cell activating factor BAFF in SS, which is associated with B cell tolerance disruption and increased autoantibody production [15]. At the same time, imbalance of miR-30b especially plays an important role in the occurrence and development of tumors [20, 21]. HLA class II genes have been identified as the strongest genetic risk factors for SS [22] and also play an important role in cancers through T cell priming, generation of strong cytotoxicity, and HLA gene methylation [23]. Additionally, chronic inflammation and tissue damage induced by SS may release cytokines such as TNF-α, IFN-γ, IL-6, and chemokines such as CXCL13 and CXCR5 which have been proved to play a role in the development of cancer through multiple mechanisms [24–27]. In summary, SS may play a vital role in the occurrence and development of cancers, and further studies are needed to explore the specific mechanism.

Mendelian randomization (MR), an epidemiological genetic approach, is applied to estimate the causal effect between exposure and outcome [28]. It uses genetic variants as instrumental variables (IVs). According to Mendel’s second law, genotypes are randomly assigned during gamete formation based on parental genotypes. Consequently, the results of MR will not be influenced by potential confounders or reverse causation [29].

In this study, using two-sample MR analysis based on the published data of genome-wide association studies (GWASs) in European population, we explored the causal effect of SS on nine types of cancers.

## Methods

### Study design

**Fig. 1:**
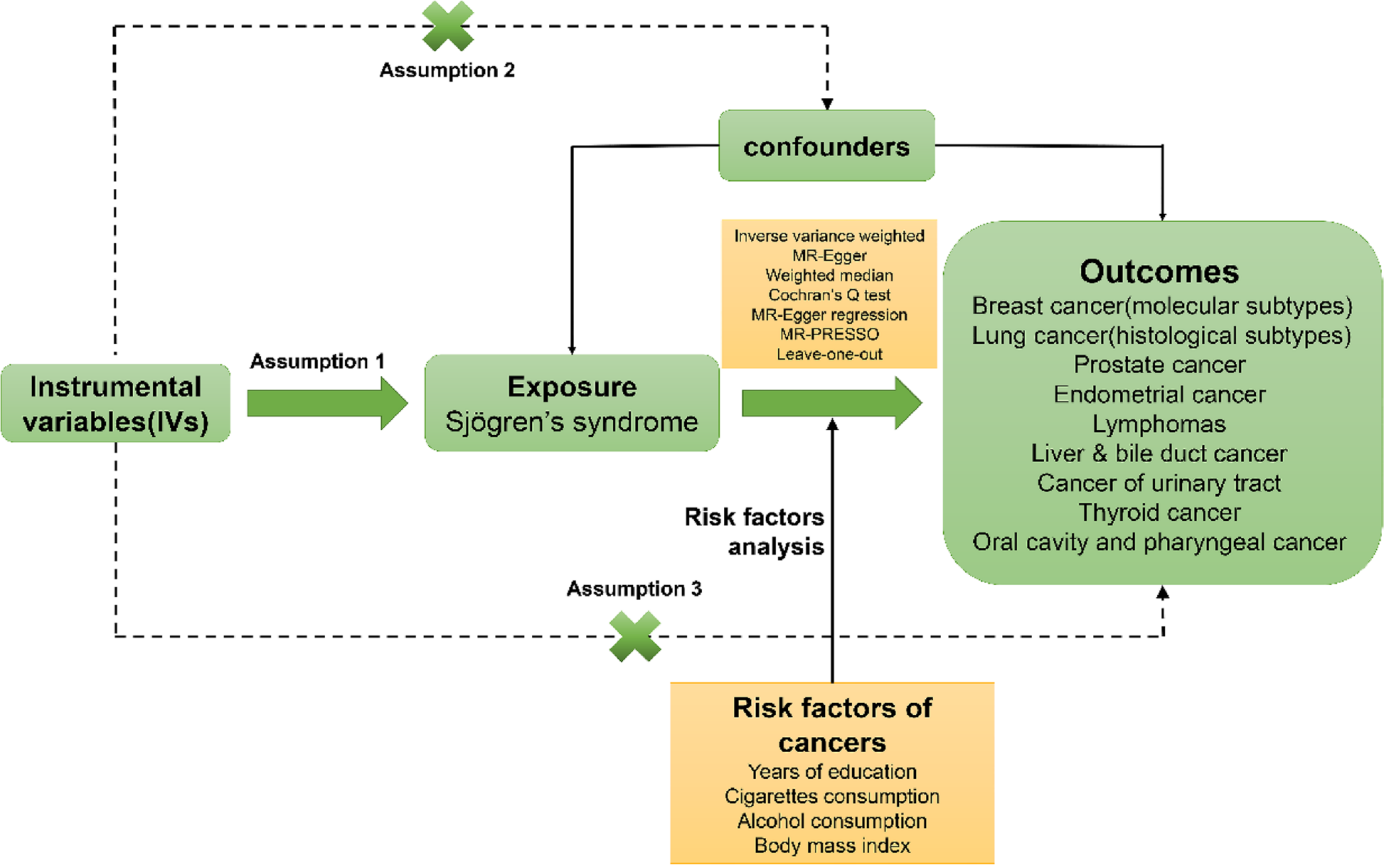
Study design of the Mendelian randomization analysis between Sjögren’s syndrome and cancers

This study evaluated whether SS was causally related to cancer risks using two-sample MR analysis based on the summary-level genetic data from previous studies and IEU OpenGWAS database in European population. Instrument variables (IVs) should follow three assumptions: (1) the IVs should be robustly associated with SS; (2) the IVs should not be associated with confounders of the SS-cancers association; (3) the IVs should influence cancers only through SS, not through any other variables (Fig. 1).

### The data source of SS

In 2013, the Sjögren’s Genetics Network (SGENE) published the first large-scale genomic study of SS of European descent, which identified seven loci [22]. In 2017, Li et al. [30] identified a new susceptibility locus of SS of European ancestry, OAS1. Carapito et al. [31] also found a new locus of European ancestry, MICA. The Sjögren’s International Collaborative Clinical Alliance (SICCA) reported the first multiethnic GWAS study of SS but did not identify new locus in European population [32]. Lastly, Khatri et al. [33] identified ten novel Sjögren’s genetic susceptibility loci of European ancestry. We combined the outcomes of these studies and obtained seventy-one single-nucleotide polymorphisms (SNPs) from nineteen loci.

### The data source of cancers

Summary statistics of multiple cancers in European population were obtained from IEU OpenGWAS database (https://gwas.mrcieu.ac.uk/) and previous studies. The GWAS summary data of overall breast cancer (228,951 participants) and its molecular subtypes including luminal A-like (28,140 participants), luminal B-like/human epidermal growth factor receptor 2 (HER2)-positive-like (22,497 participants), luminal B/HER2-negative-like (22,594 participants), HER2-enriched-like (21,533 participants), and triple-negative (22,821 participants) was extracted from the Breast Cancer Association Consortium (BCAC) [34, 35]. The data of lung cancer was obtained from the International Lung Cancer Consortium (ILCCO), including 27,209 participants were overall lung cancer, 18,336 participants were lung adenocarcinoma, and 18,313 participants were squamous cell lung cancer [36]. The summary statistics of endometrial cancer were obtained from the study of O’Mara et al. [37] (121,885 participants). The GWAS summary data on thyroid cancer was from the study of Köhler et al. [38] (1080 participants). The data of oral cavity and pharyngeal cancer was from Oncoarray oral cavity and oropharyngeal cancer [39]. The summary statistics for prostate cancer (182,625 participants), lymphomas (361,194 participants), liver and bile duct cancer (372,366 participants), and cancer of urinary tract (361,194 participants) were all extracted from UK Biobank [40] (Table 1).

**Table 1:** Details of cancers and risk factors used in the study

Trait	Consortium	N. cases	N. controls	Sample size	Year	GWAS ID	*F*-statistics
Overall breast cancer	BCAC	122,977	105,974	228,951	2017	ieu-a-1126	25693
Luminal A-like breast cancer	BCAC	7325	20,815	28,140	2020	NA	17901
Luminal B/HER2- positive-like breast cancer	BCAC	1682	20,815	22,497	2020	NA	13001
Luminal B/HER2- negative-like breast cancer	BCAC	1779	20,815	22,594	2020	NA	3052
HER2-enriched-like breast cancer	BCAC	718	20,815	21,533	2020	NA	2057
Triple-negative breast cancer	BCAC	2006	20,815	22,821	2020	NA	2054
Overall lung cancer	ILCCO	11,348	15,861	27,209	2014	ieu-a-966	25617
Lung adenocarcinoma	ILCCO	3442	14,894	18,336	2014	ieu-a-965	11397
Squamous cell lung cancer	ILCCO	3275	15,038	18,313	2014	ieu-a-967	33778
Prostate cancer	UK Biobank	9132	173,493	182,625	2021	ieu-b-4809	52234
Endometrial cancer	O’Mara et al	12,906	108,979	121,885	2018	ebi-a-GCST006464	33778
Lymphomas	UK Biobank	1752	359,442	361,194	2018	ukb-d-C_LYMPHOMA	302
Liver and bile duct cancer	UK Biobank	350	372,016	372,366	2021	ieu-b-4915	868
Cancer of urinary tract	UK Biobank	1841	359,353	361,194	2018	ukb-d-C_URINARY_TRACT	86001
Thyroid cancer	Köhler et al	649	431	1080	2013	ieu-a-1082	47320
Oral cavity and pharyngeal cancer	Oncoarray oral cavity and oropharyngeal cancer	2497	2928	5425	2016	ieu-b-89	53769
Years of schooling	SSGAC	NA	NA	766,345	2018	ieu-a-1239	34289
Cigarettes per day	GSCAN	NA	NA	337,334	2019	ieu-b-25	2870
Alcoholic drinks per week	GSCAN	NA	NA	335,394	2019	ieu-b-73	2294
Body mass index	Neale Lab	NA	NA	336,107	2017	ukb-a-248	2304

*Abbreviations*: *BCAC *Breast Cancer Association Consortium, *GSCAN *GWAS and Sequencing Consortium of Alcohol and Nicotine use, *ILCCO *International Lung Cancer Consortium, *NA *not applicable, *SSGAC *Social Science Genetic Association Consortium

### Selection of instrumental variables

The SNPs that were highly associated with exposure (*P* < 5 × 10^−8^) and with no linkage disequilibrium (*r*^2^ < 0.001 and clump window > 10,000 kb) were used as IVs. For SNPs missed in the outcome data, proxies were identified in high linkage disequilibrium (*r*^2^ > 0.8). If no appropriate proxy SNP was available, SNPs were excluded in our analysis. We also harmonized the data so that estimates of SNP-exposure and SNP-outcome were based on the same allele and remove the SNPs which were palindromic with intermediate allele frequencies. To evaluate the correlation strength and avoid bias caused by weak IVs, we calculated the *R*^2^ and *F*-statistic. *R*^2^ statistic is used to measure the proportion of variability of exposure phenotype that is explained by instruments. When the *F*-statistic was greater than 10, it was considered sufficient [41].

### The data source of risk factors for cancers

To explore whether the causal effect of SS on cancers was mediated by risk factors of cancers, we conducted the additional MR analysis. We estimated the associations between SS and generally accepted risk factors of cancers including years of education [42, 43], cigarettes consumption, alcohol consumption [44], and body mass index (BMI) [45]. The GWAS summary statistics of years of education were from the Social Science Genetic Association Consortium (SSGAC) [46]. The data of cigarettes and alcohol consumption were retrieved from the GWAS and Sequencing Consortium of Alcohol and Nicotine use (GSCAN) [47], and the data of BMI was retrieved from the Neale Lab (http://www.nealelab.is/uk-biobank) (Table 1). SS was treated as exposure, and these risk factors were taken as outcomes to perform MR analysis. Inverse variance weighted (IVW) method is used to estimate the causality. *P* < 0.05 was regarded as significant.

### Statistical analysis

To estimate the causal effects of SS on different cancers, the IVW method was as the main analysis. When all assumptions are met, IVW method has the highest statistical power [48]. MR-Egger method and weighted median method were used as complementary analyses. MR-Egger method is able to provide a consistent result under the instrument strength independent of direct effect (InSIDE) assumption [49]. Weighted median method can provide a consistent estimate even 50% IVs are invalid [50]. Heterogeneity was evaluated by Cochran’s *Q* test. The random-effect IVW method was applied in case heterogeneity was observed. MR-Egger regression test was used to assess horizontal pleiotropy. Pleiotropic effects across genetic variants are represented by the intercept [49]. We also used MR pleiotropy residual sum and outlier (MR-PRESSO) test to detect pleiotropic SNPs, correct the horizontal pleiotropy by removing the outliers, and test whether there are significant differences in the causal estimates before and after correcting outliers via distortion test [51]. Additionally, we performed “leave-one-out” analysis to test the stability of our findings.

All statistical analyses were performed in R v4.2.1 with R packages (TwoSampleMR, MR-PRESSO). A threshold of *P* < 0.0031 (0.05/16 outcomes) using the Bonferroni correction was regarded as significant evidence of an association, and 0.0031 < *P* < 0.05 was considered as suggestive evidence of an association.

## Results

### Selection of genetic instrument

After screening for close associations (*P* < 5 × 10^−8^) and independence (*r*^2^ < 0.001), we obtained twelve SNPs as instrumental variables (Supplementary Table 1). Since the sample size of different cancer types is various, the *F*-statistics ranged from 302 to 52,234 for different cancers among our study, which suggested the instruments used strongly predicted SS in our study (Table 1). For missed SNPs in outcomes, rs2304256, rs4841465, rs909685, rs1978273, rs4728142, rs12373168, and rs4969331 were chosen as proxies for rs11085725, rs11250098, rs2069235, rs2293765, rs3757387, rs7210219, and rs8071514, but other missed SNPs (rs10774671, rs2431697, rs3135394, and rs7119038) did not find their suitable proxies.

### Causal effects between SS and cancers

The main results of the MR estimates between SS and each cancer are presented in Table 2. From the results of IVW method, we detected that genetically predicted SS significantly increased the 0.10% risk of lymphomas (OR = 1.0010, 95% CI: 1.0007–1.0012, *P* = 2.26 × 10^−11^) and reduced the 0.28% risk of prostate cancer (OR = 0.9972, 95% CI: 0.9960–0.9985, *P* = 2.45 × 10^−5^) and the 5.86% risk of endometrial cancer (OR = 0.9414, 95% CI: 0.9158–0.9676, *P* = 1.65 × 10^−5^). There was suggestive evidence of causality between SS and liver and bile duct cancer (OR = 0.9999, 95% CI: 0.9997–1.0000, *P* = 0.0291), cancer of urinary tract (OR = 0.9996, 95% CI: 0.9993–0.9999, *P* = 0.0038), and oral cavity and pharyngeal cancer (OR = 1.1068, 95% CI: 1.0068–1.2167, *P* = 0.0357). For overall breast cancer, overall lung cancer, and thyroid cancer, no significant association was detected. To further explore whether SS is causally related to certain subtypes of breast cancer or lung cancer, subgroup analysis was conducted. The results showed no causal relationship between SS and breast cancer molecular subtypes (luminal A-like, luminal B/HER2-positive-like, luminal B/HER2-negative-like, HER2-enriched-like and triple-negative breast cancer) or lung cancer histological subtypes (lung adenocarcinoma and squamous cell lung cancer). MR-Egger and weighted median methods provided results of the same direction and magnitude with IVW method.

**Table 2:** The results of Mendelian randomization analysis for SS on cancers

**Cancer types**	**No. of SNPs**	**IVW (fixed)**		**MR-Egger**		**Weighted median**	
		**OR (95% CI)**	***P* value**	**OR (95% CI)**	***P* value**	**OR (95% CI)**	***P* value**
Overall breast cancer	11	0.9952 (0.9832, 1.0073)	0.4344	0.9878 (0.9364, 1.0420)	0.6620	0.9858 (0.9714, 1.0005)	0.0576
Luminal A-like breast cancer	11	1.0103 (0.9894, 1.0316)	0.3365	1.0082 (0.8706, 1.1676)	0.9153	0.9845 (1.0146, 0.9553)	0.3094
Luminal B/HER2- positive-like breast cancer	11	0.9788 (0.9344, 1.0253)	0.3654	0.8944 (0.6941, 1.1526)	0.4109	0.9506 (0.8922, 1.0128)	0.1170
Luminal B/HER2- negative-like breast cancer	11	0.9630 (0.9251, 1.0024)	0.0653	0.8975 (0.7576, 1.0633)	0.2426	0.9464 (0.8939, 1.0019)	0.0583
HER2-enriched-like breast cancer	11	0.9934 (0.9550, 1.0334)	0.7434	0.9463 (0.8080, 1.1082)	0.5105	0.9889 (0.9369, 1.0437)	0.6848
Triple-negative breast cancer	11	1.0058 (0.9404, 1.0757)	0.8662	0.9925 (0.7590, 1.2980)	0.9576	0.9704 (0.8883, 1.0601)	0.5056
Overall lung cancer	10	1.0223 (0.9743, 1.0727)	0.3690	1.0226 (0.8071, 1.2958)	0.8575	0.9797 (0.9111, 1.0535)	0.5807
Lung adenocarcinoma	10	1.0488 (0.9738, 1.1296)	0.2082	1.0716 (0.7869, 1.4593)	0.6722	1.0579 (0.9559, 1.1707)	0.2767
Squamous cell lung cancer	10	1.0343 (0.9595, 1.1149)	0.3782	1.3105 (0.8374, 2.0508)	0.2707	1.0607 (0.9390, 1.1982)	0.3432
Prostate cancer	10	0.9972 (0.9960, 0.9985)	2.45E − 05	0.9951 (0.9930, 0.9972)	0.0019	0.9961 (0.9945, 0.9978)	3.26E − 06
Endometrial cancer	12	0.9414 (0.9158, 0.9676)	1.65E − 05	0.8955 (0.8514, 0.9418)	0.0016	0.9201 (0.8876, 0.9538)	5.72E − 06
Lymphomas	12	1.0010 (1.0007, 1.0012)	2.26E − 11	1.0014 (1.0006, 1.0021)	0.0070	1.0010 (1.0007, 1.0014)	2.51E − 08
Liver and bile duct cancer	10	0.9999 (0.9997, 1.0000)	0.0291	0.9999 (0.9997, 1.0001)	0.2192	0.9998 (0.9997, 1.0000)	0.0320
Cancer of urinary tract	12	0.9996 (0.9993, 0.9999)	0.0038	0.9997 (0.9990, 1.0003)	0.3394	0.9997 (0.9993, 1.0000)	0.0531
Thyroid cancer	9	0.8832 (0.7031, 1.1094)	0.2858	1.2858 (0.4942, 3.3454)	0.6222	0.9877 (0.7223, 1.3506)	0.9383
Oral cavity and pharyngeal cancer	10	1.1068 (1.0068.1.2167)	0.0357	1.3041 (0.9847.1.7270)	0.1011	1.1764 (1.0085.1.3723)	0.0387

*Abbreviations*: *CI *confidence interval, *IVW *inverse variance weighted, *MR *Mendelian randomization, *OR *odds ratio, *SS *Sjögren’s syndrome

The scatter plots illustrated the estimated impact of instrumental variables on SS and cancers. The rising lines in the plot demonstrated the positive relationship between SS and cancers (Supplementary Fig. 1).

### Sensitivity analysis

To test the reliability of MR analysis results, we conducted sensitivity analysis. The results demonstrated significant heterogeneity in overall breast cancer, luminal A-like breast cancer, squamous cell lung cancer, lymphomas, cancer of urinary tract and oral cavity, and pharyngeal cancer (*Q*-value < 0.05) (Table 3). Thus, we corrected the heterogeneity with the random-effect IVW method. The causal effect of SS on oral cavity and pharyngeal cancer (OR = 1.1068, 95% CI: 0.9445–1.2970, *P* = 0.2098) was no longer statistically significant. The causal association between SS and overall breast cancer (OR = 0.9952, 95% CI: 0.9649–1.0264, *P* = 0.7595), luminal A-like breast cancer (OR = 1.0103, 95% CI: 0.9761–1.0457, *P* = 0.5597), squamous cell lung cancer (OR = 1.0343, 95% CI: 0.9198–1.1631, *P* = 0.5731), lymphomas (OR = 1.0010, 95% CI: 1.0005–1.0015, *P* = 0.0002), cancer of urinary tract (OR = 0.9996, 95% CI: 0.9992–1.0000, *P* = 0.0281) remained consistent with the previous results.

**Table 3:** The results of heterogeneity analysis for SS on cancers

Cancer types	Cochran’s *Q* test			IVW (random)	
	**Method**	***Q***	***Q* pval**	**OR (95% CI)**	***P* value**
Overall breast cancer	MR Egger	64.3370	1.94E − 10	0.9952 (0.9649, 1.0264)	0.7595
	IVW	65.1903	3.73E − 10		
Luminal A-like breast cancer	MR Egger	27.1406	0.0013	1.0103 (0.9761, 1.0457)	0.5597
	IVW	27.1431	0.0025		
Luminal B/HER2-positive-like breast cancer	MR Egger	16.4500	0.0581	NE	NE
	IVW	17.3952	0.0661		
Luminal B/HER2-negative-like breast cancer	MR Egger	9.9122	0.3576	NE	NE
	IVW	10.6899	0.3822		
HER2-enriched-like breast cancer	MR Egger	8.2534	0.5088	NE	NE
	IVW	8.6417	0.5664		
Triple-negative breast cancer	MR Egger	8.9561	0.4413	NE	NE
	IVW	8.9662	0.5353		
Overall lung cancer	MR Egger	13.2712	0.1029	NE	NE
	IVW	13.2712	0.1507		
Lung adenocarcinoma	MR Egger	9.8468	0.2759	NE	NE
	IVW	9.8716	0.3610		
Squamous cell lung cancer	MR Egger	19.2431	0.0136	1.0343 (0.9198, 1.1631)	0.5731
	IVW	22.0099	0.0088		
Prostate cancer	MR Egger	8.7033	0.3679	NE	NE
	IVW	15.8168	0.0708		
Endometrial cancer	MR Egger	12.0095	0.2844	NE	NE
	IVW	19.0442	0.0603		
Lymphomas	MR Egger	29.8692	0.0009	1.0010 (1.0005, 1.0015)	0.0002
	IVW	34.6995	0.0003		
Liver and bile duct cancer	MR Egger	3.9196	0.8643	NE	NE
	IVW	3.9221	0.9165		
Cancer of urinary tract	MR Egger	18.8126	0.0427	0.9996 (0.9992, 1.0000)	0.0281
	IVW	19.0671	0.0599		
Thyroid cancer	MR Egger	8.2245	0.3132	NE	NE
	IVW	8.9707	0.3448		
Oral cavity and pharyngeal cancer	MR Egger	20.4960	0.0086	1.1068 (0.9445, 1.2970)	0.2098
	IVW	25.2313	0.0027		

*Abbreviations*: *CI *confidence interval, *IVW *inverse variance weighted, *MR *Mendelian randomization, *NE *not estimate, *OR *odds ratio,  *SS *Sjögren’s syndrome

Then, the MR-Egger regression and MR-PRESSO methods were used to detect horizontal pleiotropy (Table 4), and the results of MR-Egger regression test were visualized through the funnel plots (Supplementary Fig. 2). Although the MR-Egger regression test showed prostate cancer (*P* = 0.0338) and endometrial cancer (*P* = 0.0360) existed in the horizontal pleiotropy, the MR-PRESSO method did not test the horizontal pleiotropy (*P* = 0.1589; *P* = 0.1111). From the results of the MR-PRESSO method, overall breast cancer, luminal A-like breast cancer, lymphomas, and oral cavity and pharyngeal cancer were detected in the horizontal pleiotropy (*P* < 1.00 × 10^−4^; *P* = 0.0040; *P* = 0.0147; *P* = 0.0179). After removing the outlier SNPs (rs7210219 for overall breast cancer and luminal A-like breast cancer, rs7119038 for lymphomas, rs1978273 and rs3135394 for oral cavity and pharyngeal cancer), the causal effects of SS on overall breast cancer (OR = 0.9867, 95% CI: 0.9686–1.0052, *P* = 0.1576), luminal A-like breast cancer (OR = 0.9972, 95% CI: 0.9757–1.0190, *P* = 0.7970), and lymphomas (OR = 1.0009, 95% CI: 1.0006–1.0013, *P* = 1.15 × 10^−6^) were still consistent with previous results, and the distortion tests of MR-PRESSO were not statistically significant (*P* = 0.5067; *P* = 0.1094; *P* = 0.5515), the causal effect of SS on oral cavity and pharyngeal cancer was not significant (OR = 1.0309, 95% CI: 0.9059–1.1730, *P* = 0.6447), and the distortion tests was significant (*P* = 0.0153). The leave-one-out analysis indicated that no single instrumental variable affected the causal effects of SS on cancers (Supplementary Fig. 3).

**Table 4:** The results of horizontal pleiotropy analysis for SS on cancers

Cancer types	Horizontal pleiotropy test			MR-PRESSO	
	**Intercept**	**SE**	***P* value**	***P* value**	**DT *P* value**
Overall breast cancer	0.0033	0.0094	0.7377	< 1E-04	0.5067
Luminal A-like breast cancer	0.0005	0.0186	0.9780	0.0040	0.1094
Luminal B/HER2-positive-like breast cancer	0.0231	0.0321	0.4903	0.0753	NE
Luminal B/HER2-negative-like breast cancer	0.0180	0.0214	0.4225	0.3752	NE
HER2-enriched-like breast cancer	0.0124	0.0199	0.5486	0.5845	NE
Triple-negative breast cancer	0.0034	0.0338	0.9225	0.5711	NE
Overall lung cancer	− 0.0001	0.0296	0.9978	0.1498	NE
Lung adenocarcinoma	− 0.0055	0.0386	0.8908	0.4194	NE
Squamous cell lung cancer	− 0.0597	0.0556	0.3148	0.1794	NE
Prostate cancer	0.0010	0.0004	0.0338	0.1589	NE
Endometrial cancer	0.0218	0.0090	0.0360	0.1111	NE
Lymphomas	− 0.0002	0.0001	0.2323	0.0147	0.5515
Liver and bile duct cancer	− 1.93E − 06	3.84E − 05	0.9612	0.9197	NE
Cancer of urinary tract	− 4.29E − 05	0.0001	0.7207	0.1300	NE
Thyroid cancer	− 0.0953	0.1195	0.4517	0.3302	NE
Oral cavity and pharyngeal cancer	− 0.0625	0.0460	0.2111	0.0179	0.0153

*Abbreviations*: *DT *distortion test, *MR-PRESSO *Mendelian randomization pleiotropy residual sum and outlie, *NE *not estimate, *SE *standard error, *SS *Sjögren’s syndrome

From the single SNP analysis (Table 5 and Supplementary Fig. 4), we found a potential risk SNP (rs3135394) for endometrial cancer (*P* = 1.55 × 10^−5^), prostate cancer (*P* = 1.28 × 10^−6^), and lymphomas (*P* = 4.41 × 10^−10^) through its impact on SS.

**Table 5:** The causal effects between single SNP of Sjögren’s syndrome and cancers

SNP	Endometrial cancer		Prostate cancer		Lymphomas	
	**OR (95% CI)**	***P* value**	**OR (95% CI)**	***P* value**	**OR (95% CI)**	***P* value**
rs3135394	0.9194 (0.8850, 0.9551)	1.55E − 05	0.9959 (0.9942, 0.9975)	1.28E − 06	1.0012 (1.0008, 1.0016)	4.41E − 10
rs2293765	1.0810 (0.9400, 1.2430)	0.2746	0.9946 (0.9881, 1.0012)	0.1078	0.9994 (0.9979, 1.0009)	0.4418
rs10774671	0.9054 (0.8088, 1.0135)	0.0842	0.9964 (0.9913, 1.0015)	0.1673	0.9993 (0.9981, 1.0004)	0.2095
rs7210219	1.0245 (0.8835, 1.1880)	0.7490	1.0044 (0.9974, 1.0113)	0.2183	1.0020 (1.0005, 1.0036)	0.0115
rs11250098	1.0831 (0.8457, 1.3873)	0.5271	1.0050 (0.9955, 1.0146)	0.3039	0.9995 (0.9973, 1.0017)	0.6470
rs11085725	1.0692 (0.9345, 1.2232)	0.3303	1.0030 (0.9967, 1.0093)	0.3484	1.0017 (1.0003, 1.0031)	0.0210
rs3757387	0.8952 (0.8236, 0.9731)	0.0093	0.9983 (0.9944, 1.0021)	0.3798	1.0003 (0.9994, 1.0012)	0.5284
rs8071514	0.9262 (0.7752, 1.1067)	0.3987	0.9964 (0.9883, 1.0046)	0.3937	1.0006 (0.9987, 1.0024)	0.5533
rs2069235	1.1311 (0.9531, 1.3423)	0.1585	1.0031 (0.9950, 1.0112)	0.4565	0.9996 (0.9978, 1.0015)	0.6880
rs485497	0.9882 (0.8795, 1.1103)	0.8414	0.9989 (0.9934, 1.0043)	0.6841	1.0008 (0.9996, 1.0021)	0.2029
rs7119038	0.8896 (0.7882, 1.0040)	0.0580	NE	NE	NE	NE
rs2431697	0.9632 (0.8151, 1.1383)	0.6600	NE	NE	1.0005 (0.9987, 1.0022)	0.5907

*Abbreviations*: *CI *confidence interval, *NE *not estimate, *OR *odds ratio

### Risk factors analysis

To investigate whether the causal effects of SS on cancers were violated through recognized risk factors relating to cancers, we calculated the effects of SS on years of education, cigarettes consumption per day, alcoholic consumption per week, and BMI. The results of IVW method showed that SS was not causally related to these risk factors (all *P* > 0.05) (Table 6), indicating that the causal effects between SS and cancers were not influenced by these acknowledged risk factors.

**Table 6:** The results of the risk factors analysis

Exposure	Outcomes	No. of SNPs	OR (95% CI)	*P* value
Sjögren’s syndrome	Years of schooling	10	0.9983 (0.9884, 1.0083)	0.7394
	Cigarettes per day	8	1.0055 (0.9944, 1.0168)	0.3316
	Alcoholic drinks per week	7	1.0036 (0.9997, 1.0074)	0.0683
	Body mass index	11	1.0053 (0.9956, 1.0150)	0.2844

*Abbreviations*: *CI *confidence interval, *IVW *inverse variance weighted, *MR *Mendelian randomization, *OR *odds ratio

### Causal effects of cancers on SS

From the results of IVW analysis, we found that genetically predicted lymphomas were significantly associated with SS (OR = 2.21 × 10^48^, 95% CI: 5.03 × 10^18^–9.75 × 10^77^, *P* = 0.0014). The causal relationships between genetically predicted prostate cancer, endometrial cancer, liver and bile duct cancer, cancer of urinary tract, and SS were not detected (Supplementary Table 2). Then, we conducted sensitivity analysis to test the reliability of the results. Heterogeneity was found in lymphomas and liver and bile duct cancer (Supplementary Table 3). MR-Egger regression test did not detect the horizontal pleiotropy. MR-PRESSO test showed the horizontal pleiotropy in lymphomas (Supplement Table 4). After correcting the heterogeneity and removing the outlier SNPs, the causal effect of lymphomas on SS was not significant (OR = 3.07 × 10^4^, 95% CI: 2.10 × 10^−48^–4.47 × 10^56^, *P* = 0.8661), and the causal effect of liver and bile duct cancer was still not significant (OR = 2.68 × 10^41^, 95% CI: 9.62 × 10^−59^–7.46 × 10^140^, *P* = 0.4142).

## Discussion

In this study, we explored the causal associations between SS and nine types of cancers respectively in the European population using the two-sample MR approach. The results demonstrated that SS was significantly associated with increased risks of lymphomas (OR = 1.0010, 95% CI: 1.0005–1.0015, *P* = 0.0002) and reduced risks of prostate cancer (OR = 0.9972, 95% CI: 0.9960–0.9985, *P* = 2.45 × 10^−5^) and endometrial cancer (OR = 0.9414, 95% CI: 0.9158–0.9676, *P* = 1.65 × 10^−5^). Suggestive evidence (0.0031 < *P* < 0.05) between SS and liver and bile duct cancer (OR = 0.9999, 95% CI: 0.9997–1.0000, *P* = 0.0291) and cancer of urinary tract (OR = 0.9996, 95% CI: 0.9992–1.0000, *P* = 0.0281) was detected, which indicated that there is a possible causal association between SS and live and bile duct cancer and cancer of urinary tract. The causal association was not observed in other types of cancers including overall breast cancer, overall lung cancer, thyroid cancer, and oral cavity and pharyngeal cancer. Subgroup analysis revealed that SS was not associated with the risk of different molecular subtypes of breast cancer (luminal A-like, luminal B/HER2-positive-like, luminal B/HER2-negative-like, HER2-enriched-like and triple-negative breast cancer) or the histological subtypes of lung cancer (lung adenocarcinoma and squamous cell lung cancer). At the same time, the results of risk factors analysis demonstrated no association between SS and potential risk factors (years of education, cigarettes consumption per day, alcoholic consumption per week and BMI), suggesting that the causal effect of SS on cancers we studied was not mediated by these common risk factors of cancers. We also found that a potential risk SNP (rs3135394) which is in the HLA-DRA promoter region might play an important role that determining the causal associations between SS and prostate cancer, endometrial cancer, and lymphomas. In the reverse direction, we did not find the causal relationship between cancers and SS. As far as we know, this is the first MR study to investigate the causal relationship between SS and cancers.

Among various cancers, the relationship between SS and lymphomas has been the most studied. Our findings remain consistent with previous studies showing that SS patients had an increased risk of lymphomas [52, 53]. Previous studies have shown that in the salivary glands of SS patients, immune complexes stimulate the polyclonal expansion of rheumatoid factor-reactive B cells. B cell activating factor (BAFF) stimulates the proliferation of clonal B cells when it is overproduced [54]. Suppressed IFNα leads to the survival of malignant B cells [55]. The interactions of Fms-like tyrosine kinase 3 (Flt3) and Flt3 ligand contribute to the abnormality of B cell distribution [56]. Chronic antigen stimulation and these factors promote the transformation of polyclonal to monoclonal B cell expansion [4]. The germline abnormalities of A20 (encoded by TNFAIP3) lead to the overactivation of NF-κB pathway in the continuous stimulation of B cells by autoimmunity, and the uncontrolled activation of NF-κB pathway promotes the survival of germline B cells and accumulate oncogenic mutations, which enhance the risk of lymphoma [57]. Moreover, the mutations of tumor suppressor gene p53 lead to the imbalance of cell cycle and uncontrolled cell proliferation, which promotes lymphomas development in patients with SS [58].

For cancers other than lymphomas, the conclusions among studies were inconsistent. A systematic review which included seven studies involving 22,204 SS patients demonstrated that except for lymphoma, the incidence of other malignancies was not related to SS patients [59]. A meta-analysis reviewing fourteen studies involving more than 14,523 patients with SS showed that SS was significantly associated with increased risks of thyroid cancer [60]. Recently, another meta-analysis which included twenty-five studies involving more than 47,607 SS patients found SS was significantly associated with increased risks of solid tumors, including lung, thyroid, non-melanoma skin, kidney/urinary tract, liver, and prostate cancers [61]. Observational studies are easy to be influenced by potential residual bias, insufficient power owing to small sample size, and reverse causality, which may lead to the controversial conclusions. MR designs can simulate randomized controlled trails and avoid reverse causality and confounding bias of observational studies, which are more practical to reveal causal association. Although large-scale randomized controlled trails (RCTs) are advocated for exploring the causal association between certain exposure and diseases, MR could provide reliable evidence for researchers to decide whether the time-consuming and costly RCTs should be further conducted. Therefore, our study used MR method with a large sample size to explore the causal effect of SS on cancers.

Concerning breast cancer, lung cancer, thyroid cancer, and oral cavity and pharyngeal cancer, we obtained the similar results to other studies based on the European population [6, 8, 9, 62]. Causal relationship between SS and these cancers were not proven yet.

Interestingly, we observed significant associations between SS and reduced risks of prostate cancer and endometrial cancer. For prostate cancer, some researches did not observe the difference in the incidence risk of prostate cancer compared with controls [6, 53, 63]. Wang et al. [64] reported that in Taiwanese population, a significant correlation was found between SS and the elevated risk of prostate cancer. However, the conflicting results may be attributed to different genetic and environment background of different ethnic groups [2]. So, further studies based on a larger sample size in European population or comparative analysis among different ethnic groups are needed. For endometrial cancer, our study found a reduced risk of endometrial cancer in patients with SS (OR = 0.9414, 95% CI: 0.9158–0.9676). Two observational studies also suggested reduced incidence of carcinoma of uterine corpus among patient with SS [6, 9], but these differences did not reach statistical significance. Lower level of dehydroepiandrosterone-sulfate pro-hormone is detected in SS patients compared to healthy controls, and the conversion of dehydroepiandrosterone to dihydrotestosterone and estrogen is impaired in patients with SS [65]. This abnormity leads to a lower estrogen exposure in patients with SS compared to their controls [66]. Endometrial cancer has been proved to be driven by estrogen [67], which may explain why SS patients tend to have lower incidence of endometrial cancer. HLA-DRA, a candidate risk gene, might provide a novel point for the causal associations between SS and prostate cancer, endometrial cancer, and lymphomas. HLA-DRA encodes the HLA-DR alpha chain. HLA-DR is known to help presenting tumor-associated antigens (TAA) to T cells, which then triggers series of immune response in the tumor microenvironment [68]. Several studies have explored the association between HLA-DRA and cancers and suggested a role HLA-DRA played during the development of multiple cancers including diffuse large B cell lymphoma [69], endometrial cancer [70], prostate cancer [71], hepatocellular carcinoma [72], cervical cancer [73], bladder cancer [74], colorectal cancer [75], and ovarian cancer [76]. Additionally, HLA-DRA may represent as a predictive marker for cancer risk and prognosis. SNP rs3135394 is located in the HLA-DRA promoter region, which encodes the HLA-DR alpha chain. From the single SNP analysis, we found rs3135394 might play a vital role in the causal relationship between SS and endometrial cancer (*P* = 1.55 × 10^−5^), prostate cancer (*P* = 1.28 × 10^−6^), and lymphomas (*P* = 4.41 × 10^−10^), but further studies on the mechanism of rs3135394 are still warranted.

We also revealed the suggestive evidence of associations between SS and reduced risks of cancer of urinary tract and liver and bile duct cancer. For urinary tract cancer, Theander et al. [53] and Treppo et al. [63] reported that there is no association between SS and kidneys/urinary tract cancer in European population. Zhou et al. [77] illustrated that SS was associated with an increased risk of urinary tract cancer in Chinese population. So, further studies focused on urinary tract cancer and based on a larger sample size in European population, or comparative analysis among different ethnic groups is needed. A few studies showed that SS was correlated with an increased risk of liver cancer [64], but our study suggested lower risk for liver and bile duct cancer among patients with SS. MR approach is based on the GWAS data, of which the sample size is hundreds of thousands of or even millions of genetic variants, much larger than the observational studies we referred to. In addition, the study design and analytical principles of observational studies and MR are different, and the results of observational studies tend to be affected by reverse causality and confounding variables, which may lead to biased associations or conclusions. These aspects may explain the discordance between the observational studies and our MR analysis. Thus, further studies are warranted.

Immunosuppressive therapy is the major systemic treatment for SS [78]. However, differences in malignancy rates have been observed between patients with autoimmune diseases who used certain immunosuppressive agents and the general population [79, 80]. It is possible that immunosuppressive agents used for long-term therapy of SS may mediate the causal relationship between SS and cancers. Methotrexate, an immunoregulatory and anti-inflammatory agent, is often used to treat SS [81]. In several retrospective cohort studies, patients with rheumatoid arthritis or psoriatic arthritis who use methotrexate have been linked to an increased risk of skin cancer [82–84]. Rituximab is a chimeric anti-CD20 monoclonal antibody, which helps to relieve the sicca symptoms of SS by depleting B cells and impairing the number and function of T cells [85]. A retrospective cohort study in Sweden showed no difference in cancer risk between multiple sclerosis patients using rituximab and the general population [86]. Leflunomide is another immunosuppressive and anti-inflammatory agent use in SS treatment. However, there is currently no evidence of correlation between leflunomide administration and higher cancer incidence. Some studies even suggested anti-cancer potential of leflunomide [87]. It remains unclear whether immunosuppressive agents or antibodies used for SS treatment affects the casual relationship between SS and carcinogenesis.

Several advantages exist in our study. First, it is the first MR study to evaluate the potential causal associations between SS and certain cancers. Our study strictly followed the three major assumptions of MR analysis and could prevent the influence of potential confounders and reverse causality to a certain extent. Second, we included the most comprehensive SS-related SNPs to date, which could better explain genetic variants of SS. At the same time, the sample sizes of nine types of cancers vary from 1080 to 372,373 and are much larger than previous observational studies, which could provide adequate statistical power to assess the causality. Moreover, since we noticed that different molecular or histologic subtypes of cancer may share distinct biological characteristics based on both scientific researches or clinical observations, subgroup analysis were conducted on breast cancer and lung cancer, and the results could help to reach a deeper understanding of the causal relationship between SS and these cancers.

There are also limitations in our study. First, the participants of our study were all of the European descent. Whether conclusions of our study could be applied to other populations or regions is uncertain. So, further studies based on other populations or regions are needed. Second, the influence of potential pleiotropy on MR studies is difficult to be eliminated completely. In this study, we observed the horizontal pleiotropy in prostate cancer and endometrial cancer through MR-Egger regression test, but MR-PRESSO method did not test the horizontal pleiotropy and detect pleiotropic SNPs in them. In addition, though we included the most comprehensive GWASs summary data of SS so far, these SNPs only explained a small part of variants of SS. It is possible that other unknown SS-related SNPs could also play an important role in cancers. Thus, it is necessary to conduct new GWASs to detect novel SS-related SNPs.

## Conclusions

This is the first study to revealed the causal effect of Sjögren’s syndrome on cancers using MR analysis. Sjögren’s syndrome was causally associated with risks of some cancers such as lymphomas, prostate cancer, endometrial cancer, liver and bile duct cancer, and urinary tract cancer, indicating that Sjögren’s syndrome may play a vital role in the occurrence of these malignancies. Further large-scale studies are warranted to verify our results and the underlying mechanisms should be further explored.

### Supplementary Information

**Additional file 1: Supplementary Table 1.** Details of SNPs selected as instrumental variables for Mendelian randomization analysis. **Supplementary Table 2.** The results of the causal effects of cancers on Sjögren’s syndrome. **Supplementary Table 3.** The results of heterogeneity analysis in inverse direction analysis. **Supplementary Table 4.** The results of horizontal pleiotropy analysis in inverse direction analysis. **Supplementary Fig. 1.** The scatter plots of the effects of genetic instruments on Sjögren’s syndrome against their effects on cancers. **Supplementary Fig. 2.** The funnel plots of the causal effect of Sjögren’s syndrome on cancers. **Supplementary Fig. 3.** Leave-one-out analysis of the causal effect of Sjögren’s syndrome on cancers. **Supplementary Fig. 4.** The forest plots of the causal effect of Sjögren’s syndrome on cancers

## Data Availability

The data used in this study was obtained from public databases and previous studies. Data sources and handing of these data were described in the Materials and Methods. Further information is available from the corresponding author upon request.

## Abbreviations

CI: Confidence interval
GWASs: Genome-wide association studies
IVs: Instrumental variables
IVW: Inverse variance weighted
MR: Mendelian randomization
MR-PRESSO: MR pleiotropy residual sum and outlier
OR: Odds ratio
SNP: Single-nucleotide polymorphisms
SS: Sjögren’s syndrome
