# Supplementary material for: Causal associations of Sjögren’s syndrome with cancers: a two-sample Mendelian randomization study
Source: Arthritis Res Ther. 2023 Sep 15;25:171. doi: 10.1186/s13075-023-03157-w (PMC10503000; doi:10.1186/s13075-023-03157-w)
Supplement: Supplementary file 1 — Additional file 1: Supplementary Table 1. Details of SNPs selected as instrumental variables for Mendelian randomization analysis. Supplementary Table 2. The results of the causal effects of cancers on Sjögren’s syndrome. Supplementary Table 3. The results of heterogeneity analysis in inverse direction analysis. Supplementary Table 4. The results of horizontal pleiotropy analysis in inverse direction analysis. Supplementary Fig. 1. The scatter plots of the effects of genetic instruments on Sjögren’s syndrome against their effects on cancers. Supplementary Fig. 2. The funnel plots of the causal effect of Sjögren’s syndrome on cancers. Supplementary Fig. 3. Leave-one-out analysis of the causal effect of Sjögren’s syndrome on cancers. Supplementary Fig. 4. The forest plots of the causal effect of Sjögren’s syndrome on cancers [file 13075_2023_3157_MOESM1_ESM.pdf]

Supplemental material for

Causal Associations of Sjögren's Syndrome with Cancers: A Two-sample Mendelian Randomization Study

Supplementary Table 1. Details of SNPs selected as instrumental variables for Mendelian randomization analysis.

Supplementary Table 2. The results of the causal effects of cancers on Sjögren's syndrome.

Supplementary Table 3. The results of heterogeneity analysis in inverse direction analysis.

Supplementary Table 4. The results of horizontal pleiotropy analysis in inverse direction analysis.

Supplementary Figure 1. The scatter plots of the effects of genetic instruments on Sjögren's syndrome against their effects on cancers.

Supplementary Figure 2. The funnel plots of the causal effect of Sjögren's syndrome on cancers.

Supplementary Figure 3. Leave-one-out analysis of the causal effect of Sjögren's syndrome on cancers.

Supplementary Figure 4. The forest plots of the causal effect of Sjögren's syndrome on cancers.

Table S1. Details of SNPs selected as instrumental variables for Mendelian randomization analysis.

SNP	Effect allele	Other allele	EAF	P value	Beta	SE
rs2293765	A	C	0.5586	5.53E-14	0.2151	0.0286
rs2431697	C	T	0.4314	3.33E-09	-0.1863	0.0315
rs11250098	A	G	0.4851	6.89E-12	0.1484	0.0216
rs7210219	C	T	0.2406	2.40E-10	-0.2485	0.0392
rs8071514	A	C	0.5477	1.64E-08	-0.1744	0.0309
rs11085725	C	T	0.7306	7.17E-13	-0.2485	0.0346
rs2069235	A	G	0.2992	5.06E-10	0.1906	0.0307
rs3135394	G	A	0.0795	5.22E-113	1.2585	0.0557
rs3757387	C	T	0.4238	2.73E-19	0.3646	0.0406
rs485497	A	G	0.5348	1.17E-10	0.2624	0.0407
rs7119038	A	G	0.7704	1.10E-08	-0.3011	0.0527
rs10774671	A	G	0.6481	2.59E-09	-0.2877	0.0483

Abbreviations: EAF, effect allele frequency; SE, standard error; SNP, single-nucleotide polymorphisms.

Table S2. The results of the causal effects of cancers on Sjögren's syndrome.

Cancer Types	No. of SNPs	IVW(FIXED)		MR-Egger		Weighted median	
		OR (95% CI)	P value	OR (95% CI)	P value	OR (95% CI)	P value
Prostate cancer	10	0.9365(2.46E-03,3.56E+02)	0.9827	1.40E+3(5.87E-09,3.32E+14)	0.6027	4.77E-02(1.01E-05,2.25E+02)	0.4809
Endometrial cancer	5	1.0142(0.5931,1.7343)	0.9589	0.7564(0.0708,8.0808)	0.8322	1.0331(0.5300,2.0140)	0.9237
Lymphomas	4	2.21E+48(5.03E+18,9.75E+77)	0.0014	4.41E+93(7.26E-140, Inf)	0.5130	1.45E+29(1.38E-36,1.53E+94)	0.3794
Liver & bile duct cancer	6	2.68E+41(6.71E-26,1.07E+108)	0.2228	1.6E+38(0, Inf)	0.8651	4.74E+02(5.25E-90,4.29E+94)	0.9545
Cancer of urinary tract	3	4.04E+23(2.53E-16,6.44E+62)	0.2379	1.85E+257(0, Inf)	0.5491	6.71E+17(1.48E-30,3.05E+65)	0.4635

Abbreviations: CI, confidence interval; Inf, Infinity; IVW, inverse variance weighted; MR, Mendelian randomization; OR, odds ratio.

Table S3. The results of heterogeneity analysis in inverse direction analysis.

Cancer Types	Cochran's Q test			IVW(random)	
	method	Q	Q_pval	OR (95% CI)	P value
Prostate cancer	MR Egger	14.5810	0.068	NE	NE
	IVW	15.1823	0.086		
Endometrial cancer	MR Egger	2.8744	0.411	NE	NE
	IVW	2.9365	0.569		
Lymphomas	MR Egger	19.3011	6.44E-05	2.21E+48(9.56E-31,5.13E+126)	0.2266
	IVW	20.9655	1.07E-04		
Liver & bile duct cancer	MR Egger	11.1467	0.025	2.68E+41(9.62E-59,7.46E+140)	0.4142
	IVW	11.1474	0.049		
Cancer of urinary tract	MR Egger	0.2498	0.617	NE	NE
	IVW	0.8578	0.651		

Abbreviations: CI, confidence interval; IVW, inverse variance weighted; MR, Mendelian randomization; NE, not estimate; OR, odds ratio.

Table S4. The results of horizontal pleiotropy analysis in inverse direction analysis.

Cancer Types	Horizontal pleiotropy test			MR-PRESSO	
	Intercept	SE	P value	P value	DT P value
Prostate cancer	-0.0613	0.1068	0.582	0.122	NE
Endometrial cancer	0.0366	0.1470	0.819	0.573	NE
Lymphomas	-0.1512	0.3641	0.718	0.046	<1E-04
Liver & bile duct cancer	0.0043	0.2687	0.988	0.076	NE
Cancer of urinary tract	-0.8481	1.0876	0.578	NE	NE

Abbreviations: DT, distortion test; MR-PRESSO, Mendelian randomization pleiotropy residual sum and outlie; NE, not estimate; SE, standard error.

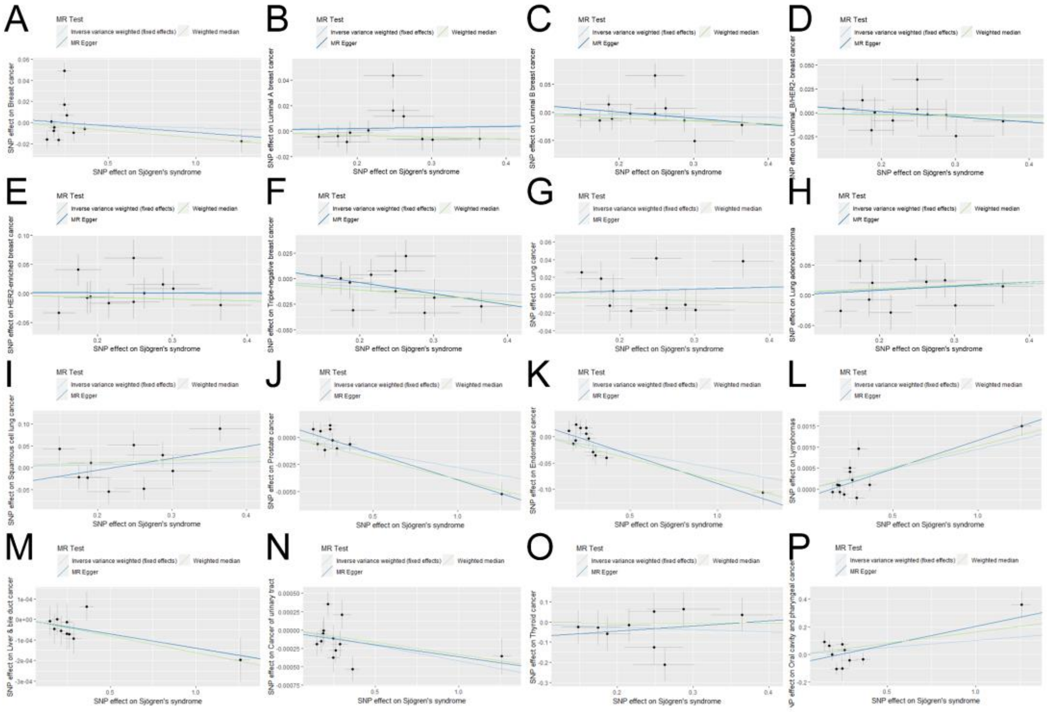

Figure S1. The scatter plots of the effects of genetic instruments on Sjögren's syndrome against their effects on cancers. The effect of Sjögren's syndrome on overall breast cancer(A), luminal A-like breast cancer(B), luminal B/HER2- positive-like breast cancer(C), luminal B/HER2- negative-like breast cancer(D), HER2-enriched-like breast cancer(E), triple-negative breast cancer(F), overall lung cancer(G), lung adenocarcinoma(H), squamous cell lung cancer(I), prostate cancer(J), endometrial cancer(K), lymphomas(L), liver & bile duct cancer(M), cancer of urinary tract(N), thyroid cancer(O), oral cavity and pharyngeal cancer(P). MR, Mendelian randomization; SNP, single-nucleotide polymorphisms.

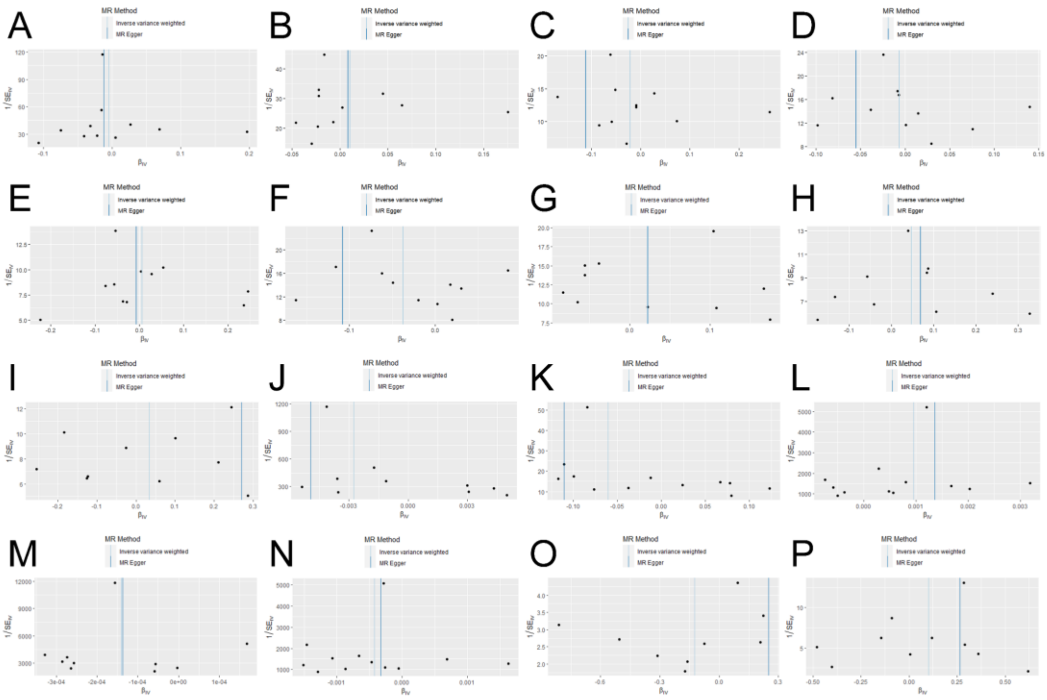

Figure S2. The funnel plots of the causal effect of Sjögren's syndrome on cancers. The effect of Sjögren's syndrome on overall breast cancer(A), luminal A-like breast cancer(B), luminal B/HER2- positive-like breast cancer(C), luminal B/HER2- negative-like breast cancer(D), HER2-enriched-like breast cancer(E), triple-negative breast cancer(F), overall lung cancer(G), lung adenocarcinoma(H), squamous cell lung cancer(I), prostate cancer(J), endometrial cancer(K), lymphomas(L), liver & bile duct cancer(M), cancer of urinary tract(N), thyroid cancer(O), oral cavity and pharyngeal cancer(P). β_{IV}, Mendelian randomization estimates of Sjögren's syndrome effect on cancers; SE_{IV}, standard error of Mendelian randomization estimates.

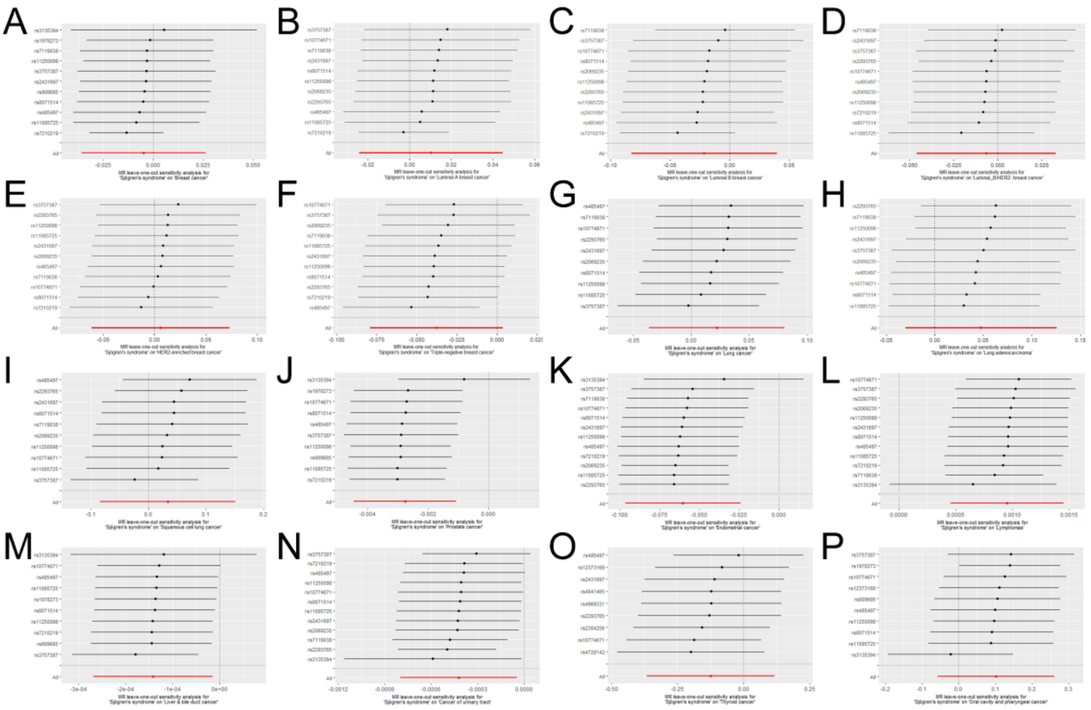

Figure S3. Leave-one-out analysis of the causal effect of Sjögren's syndrome on cancers. The effect of Sjögren's syndrome on overall breast cancer(A), luminal A-like breast cancer(B), luminal B/HER2- positive-like breast cancer(C), luminal B/HER2- negative-like breast cancer(D), HER2-enriched-like breast cancer(E), triple-negative breast cancer(F), overall lung cancer(G), lung adenocarcinoma(H), squamous cell lung cancer(I), prostate cancer(J), endometrial cancer(K), lymphomas(L), liver & bile duct cancer(M), cancer of urinary tract(N), thyroid cancer(O), oral cavity and pharyngeal cancer(P). MR, Mendelian randomization.

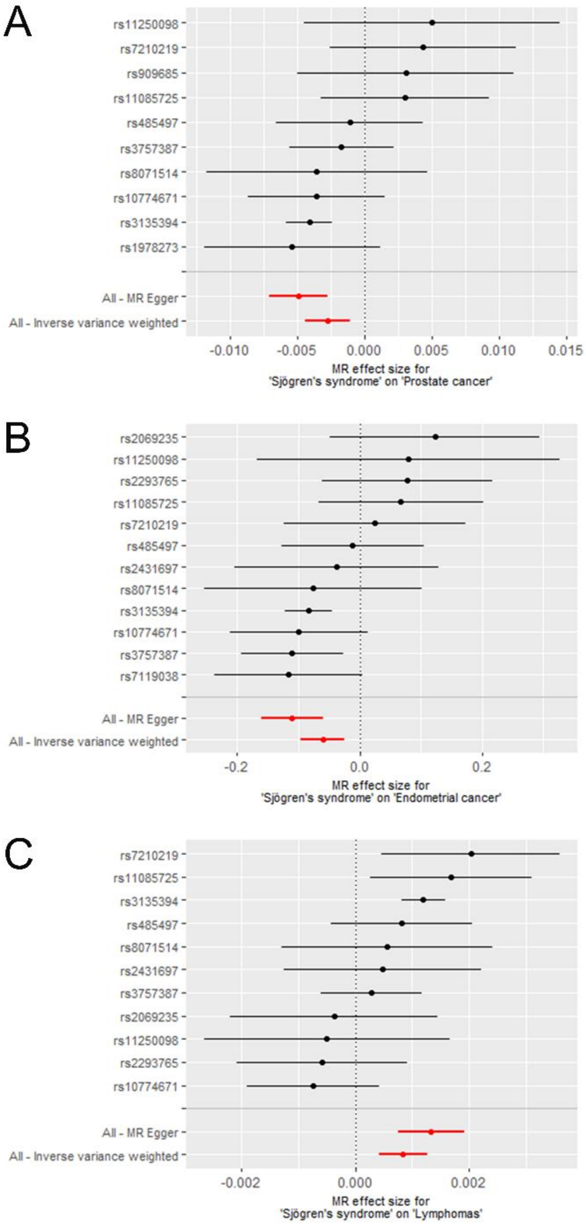

Figure S4. The forest plots of the causal effect of Sjögren's syndrome on cancers. The effect of Sjögren's syndrome on prostate cancer(A), endometrial cancer(B), lymphomas(C). MR, Mendelian randomization.
